# Antibody–Drug Conjugates for Cancer Therapy

**DOI:** 10.3390/biomedicines4030014

**Published:** 2016-07-11

**Authors:** Adam C. Parslow, Sagun Parakh, Fook-Thean Lee, Hui K. Gan, Andrew M. Scott

**Affiliations:** 1 Tumour Targeting Laboratory, Olivia Newton-John Cancer Research Institute, Heidelberg, Victoria 3084, Australia; adam.parslow@onjcri.org.au (A.C.P.); sagun.parakh@onjcri.org.au (S.P.); ft.lee@onjcri.org.au (F.-T.L.); hui.gan@onjcri.org.au (H.K.G.); 2 School of Cancer Medicine, La Trobe University, Heidelberg, Victoria 3084, Australia; 3 Department of Medical Oncology, Olivia Newton-John Cancer and Wellness Centre, Austin Health, Heidelberg, Victoria 3084, Australia; 4 Department of Medicine, University of Melbourne, Melbourne 3010, Australia; 5 Department of Molecular Imaging and Therapy, Austin Health, Heidelberg, Victoria 3084, Australia

**Keywords:** ADC, antibody–drug conjugate, resistance, monoclonal antibodies, cancer, immunotherapy

## Abstract

Antibody–drug conjugates (ADCs) take advantage of the specificity of a monoclonal antibody to deliver a linked cytotoxic agent directly into a tumour cell. The development of these compounds provides exciting opportunities for improvements in patient care. Here, we review the key issues impacting on the clinical success of ADCs in cancer therapy. Like many other developing therapeutic classes, there remain challenges in the design and optimisation of these compounds. As the clinical applications for ADCs continue to expand, key strategies to improve patient outcomes include better patient selection for treatment and the identification of mechanisms of therapy resistance.

## 1. Introduction

The search for the “magic bullet” to selectively deliver a cytotoxic agent to the site of a cancerous cell has been the goal of clinical oncology for more than 100 years [1]. Monoclonal antibody (mAb) therapy is arguably one of the most successful treatment strategies for patients with haematological and solid tumour malignancies [2]. Antibody-based therapies have therapeutic effects through a range of mechanisms, including altering antigen or receptor function and signalling, inducing complement-dependent cytotoxicity (CDC) or antibody-dependent cytotoxicity (ADCC) [3]. Even with more than 20 monoclonal antibodies approved for therapeutic use in cancer patients, further development is required to increase their effectiveness and reduce their toxicity [4]. Antibody–drug conjugates combine the ability to link a cytotoxic payload to a monoclonal antibody which specifically recognises a cellular surface antigen and deliver a toxic payload directly into the target cell [5]. The development of an effective ADC therapy is influenced by the interplay of the three main structure elements of the molecule: the antibody, the linker, and the covalently attached cytotoxic agent [6]. Depending of the target antigen in question, the use of monoclonal antibodies reduces the off target effects by limiting the exposure of normal tissues to the payload compared with conventional systemic therapies [2]. The ability to uniquely deliver a cytotoxic payload to a tumour cell provides a tenable excitement to the cancer biology field.

This review will provide a summary of the key factors leading to the emerging clinical success of ADCs and identify the challenges and advancements that have defined this exciting area of cancer therapy.

## 2. Selecting an Appropriate Target

**Figure 1:**
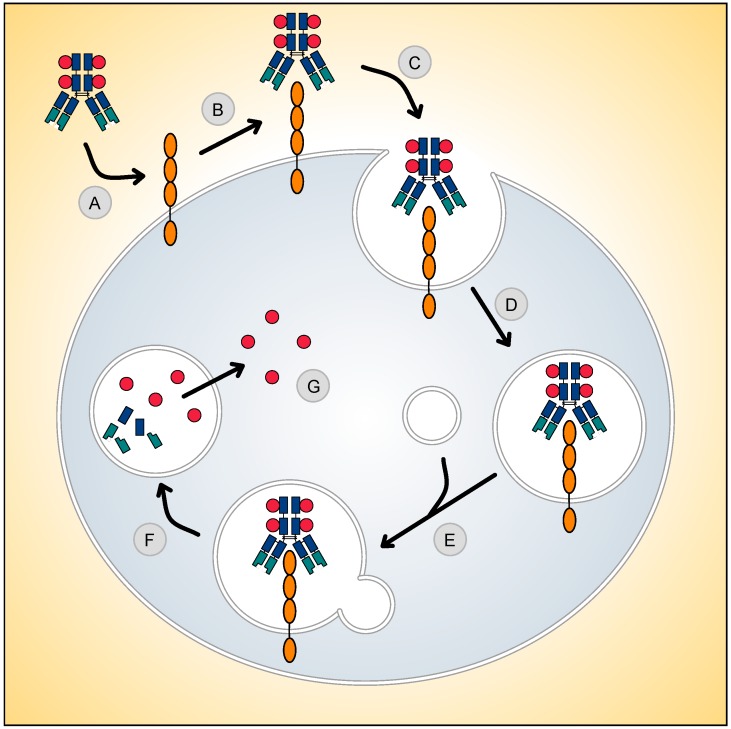
Mechanism of antibody–drug conjugate (ADC) action. (**A**) An ideal antigen target for ADC therapy is accessible via the circulation. (**B**) Following antigen binding, (**C**) the antigen-ADC complex is rapidly internalised into (**D**) endosomal vesicles and is processed along the (**E**) endosomal-lysosomal pathway. (**F**) In this acidic and proteolytic rich environment, degradation occurs, (**G**) resulting in the intracellular release of cytotoxic compound.

The appropriate selection of the antigen-binding site is a critical developmental step for the eventual success of an antibody–drug conjugate. As such, the most effective antigens share certain characteristics. The monoclonal antibody selected, as the basic structural element of the ADC, binds to a target antigen present on the surface of the cell that is accessible via the bloodstream. Following binding, the complex must be rapidly internalised, allowing the release the cytotoxic agent within the tumour cell (Figure 1) [7,8]. Ideally, the antigen should be well-characterised, proportionally abundant, and accessible on tumour cells compared to surrounding normal cell populations [9,10]. This is to allow the preferential binding and delivery of the ADC to malignant populations, reducing the potential for toxicity to normal cells. The antibody selected should have a high affinity for its target, increasing the potential for internalisation of the cytotoxic agent. Heterogeneity throughout the tumour population should be avoided, and antigens that shed and are abundant in the circulation should be avoided. The uptake of the ADC is limited by the rate of this target antigen-ADC complex internalisation [11]. The rapid uptake of the ADC also reduces extracellular payload release. The ability to identify an appropriate antigen target that contains all these characteristics is, in practice, difficult. The selection of antigen in further complicated by the constant evolutionary pressure placed on cancer cell populations during treatment. Ideally, the target antigen should not be downregulated post-treatment to maintain cellular sensitivity to the therapy.

## 3. Antibody–Drug Conjugation

The chemical conjugation of the antibody and the cytotoxic payload has a major influence on the pharmacokinetics, selectivity, and the therapeutic index of ADC-based therapies [12]. A linker is the required covalent connection between the cytotoxic compound and the antibody. The linker influences the stability and drug to antibody ratio (DAR) of the therapeutic agent. These parameters are critical for the overall success of an ADC-based design. With the high potency of the payloads chosen, linkers must be stable within the bloodstream to limit early payload release and allow tumour site targeting to occur. However, this stability must be context-dependent: once inside the cell, these linkers must be efficient in releasing the payload to induced the cytotoxic effect [13].

Conventional ADC conjugation involves methods of alkylation of reduced interchain disulphides and alkylation or acylation of lysine residues [9]. These conjugation methods generate heterogeneous mixtures of ADCs with variable drug per antibody ratios (DARs). This varies between zero and eight conjugated payloads per antibody [14]. T-DM1, for example, has on average a DAR of 3.5 [15]. The position and number of payloads bound to the antibody can have profound effects on the binding to the antigen, the aggregation of the ADC, the pharmacokinetic characteristics of the antibody construct, and even the safety profile of the ADC [16].

Improving the antibody site for linker conjugation has been greatly enhanced through advancements in protein engineering. Antibody site-specific alterations enhance linker conjugation and result in homogeneous ADCs. These alterations can be mediated through enzymatic conjugation or insertion of reactive cysteine or chemoselective functional groups of unnatural amino acid residues into the antibody protein sequence [17]. The covalent connection between drug and antibody has been traditionally mediated through the thiol group of cysteine or the epsilon amino group of lysine residues [18].

The majority of ADCs in clinical trials attach the linker to a cysteine of the antibody. Cysteines are present within the intra and interchain disulphide bridges within an antibody. The presence of 4 interchain disulphide bridges within in an IgG1 antibody typically results in only eight possible conjugation sites. This reduces potential ADC heterogeneity resulting from these reactions. The linking to antibody-derived cysteine is commonly mediated through maleimide-type linkers. The two most common are maleimidocaproyl (mc) and maleimidomethyl cyclohexane-1-carboxylate (mcc). An example is the vc-MMAE linker-payload, which contains a mc spacer attached to the valine-citrulline (vc) dipeptide, which in turn is linked to the self-immolative spacer, para-amino benzyloxycarbonyl (PABC), which is attached to a monomethylauristatin E (MMAE) payload (mc-vc-PABC-MMAE). The mc spacer allows for the lysosomal vc processing via cathepsin B, liberating an unstable PABC-MMAE product, from which PABC disintegrates leaving a chemically unmodified MMAE [19]. There are four lysine-based linkers currently used for ADCs in the clinic: *N*-succinimidyl-4-(2-pyridyldithio) butanoate (SPDB), *N*-succinimidyl-4-(2-pyridyldithio)-2-sulfo butanoate (sulfo-SPDB), maleimidomethyl cyclohexane-1-carboxylate (MCC), and hydrazone [20]. In comparison to the limited number of cysteines, an IgG1 contains approximately 90 lysines, 30 of which are available for conjugation. This is advantageous, as no antibody structural changes are required, but they increase the potential for ADC heterogeneity during production.

Linker chemistry can be broadly defined into two groups: either cleavable or non-cleavable linkers. Cleavable linkers can be sub-characterised as either: acid-sensitive, protease-sensitive, or glutathione-sensitive. A cleavable linker takes advantage of the intracellular environment of target cells. Acid-liable linkers, such as hydrazone, require the low pH of endosomes and lysosomes to trigger its hydrolysis and release of toxic payload. One such example, hydrazone, was used as the linker for gematuzumab ozogamicin, an ADC combining an anti-CD33 antibody to a calicheamicin-derived cytotoxic. However, a low serum plasma stability of approximately 48–72 h was observed [21].

Peptide linkers of the protease sensitive class are more stable than an acid-sensitive-based linker and has been successfully used in the clinically approved brentuximab vedotin linking the payload monomethylauristatin E (MMAE) to the anti-CD30 antibody [19]. Peptide linkers, such as the valine-citrulline (vc) dipeptide, contain an engineered lysosomal-specific protease (i.e., cathepsin B) cleavage site, allowing for the chemically unmodified release of cytotoxic payload from the lysosome compartment. Taking advantage of the increased glutathione concentration within tumour cells, the reducible, disulphide bond-based glutathione sensitive linkers are a third major sub-category of cleavable linkers [9,22]. However, low levels of glutathione is also present within the circulation (2.8 ± 0.9 µM) [23]. Increasing levels of disulphide steric hindrance through the insertion of methyl groups has proportionally reduced their untimely release within the circulation [24]. Lorvotuzumab mertansine (IMGN901) is the most clinically advanced ADC employing a hindered disulphide linker. This anti-CD56 targeting ADC with a maytanisinoid (DM1) payload has shown efficacy against Merkel cell carcinoma in early phase trials and has been granted orphan status by the U.S. Food and Drug Administration (FDA) [25].

The second class of ADC linking strategies involves the use of non-cleavable thioether linkers that require the post-internalisation degradation of the ADC complex within the lysosomal and endosomal compartments. This disintegration results in the release of a cytotoxic agent still attached with the linker and an amino acid from the original antibody attachment site [26]. Ado-trastuzumab emtansine (T-DM1) has successfully used a non-cleavable linker to combine a maytansinoids toxic to the anti-HER2 trastuzumab antibody [27]. Non-cleavable linkers have improved stability in the bloodstream, longer half-lives, and hence reduced risk of off-target toxicity [28]. T-DM1, for example, has a serum stability of more than three days [29]. The success of each linking strategy is context-dependent, with advantages and disadvantages for each. The conjugation method used to combine the linker to exposed residues on the surface of antibody is another pivotal step in the manufacture of a successful ADC.

## 4. Antibody–Drug Conjugate Payloads

The first generation of ADC payloads were developed around clinically approved cytotoxic agents. The well-known safety and efficacy of agents such as doxorubicin, 5-fluorouracil, and methotrexate was seen as advantageous; however, these agents did not achieve clinical benefit as ADCs due to their moderate cytotoxic potential, lack of selectivity, and low intracellular drug concentration [11]. Subsequent approaches have involved identifying drugs that were suitable for antibody conjugation and would deliver an effective cytotoxic dose, even with small efficiencies in intracellular delivery. The two most commonly used cytotoxic payloads focus on either the tubulin-targeting anti-mitotic agents or DNA-damaging drugs. The majority of payloads in current clinical development reside in the broad class of tubulin-targeting anti-mitotic agents, the maytansinoids and auristatins. These agents inhibit spindle and microtubule dynamics during interphase, resulting in G2/M mitotic arrest [30,31]. Payloads that target the microtubule network may not be limited to inhibiting cellular division and may also inhibit intracellular trafficking of proteins essential for cancer cell function [32,33]. This is particularly important for ADC-based targeting of tumour initiating cells (TIC) or quiescent cell populations.

The search for the next generation of cytotoxic payloads continues, with one approach being the development of DNA-damaging agents such as pyrrolobenzodiazepine (PBD). PBD dimers covalently bind to the minor groove of DNA and display promising pre-clinical results including efficacy 10 times lower than anti-microtubule alternatives [34,35]. The HER2-targeting ADC, SYD985, has shown promising results in early Phase I trials, with a new payload class of DNA-alkylating duocarmycins. This cleavable linker-duocarmycin payload, valine-citrulline-seco Duocarmycin hydroxybenzamide azaindole (vc-seco-DUBA), is rapidly degraded in the plasma, potentially increasing the maximum tolerated doses for this ADC class [36,37]. Potent anthracycline analogues are also showing important cytotoxicity for in vivo models conditionally resistant to MMAE-based ADCs [38]. Other agents under investigation include the RNA polymerase II binding α-amanitin [39] which can induce inhibition of DNA transcription and apoptosis; as well as the tubulin polymerisation inhibiting tubulysins [40].

In addition to the direct killing of cells that are target antigen positive, some ADCs are also able to kill target antigen negative neighbouring cell populations, known as a bystander killing effect [41]. The effective clinical response of the anti-CD30 MMAE delivering brentuximab vedotin is suggested to be partially induced by the diffusion of MMAE into the surrounding tumour environment [42]. The 3% CD30-positive target expression in lymphoma cells in a highly responsive brentuximab vedotin patient is highly suggestive of this hypothesis [43]. The membrane permeable PBD and MMAE payloads are able to induce potent bystander killing mechanisms compared with the more hydrophilic and thus less permeable MMAF molecule, which has reduced bystander killing potential [26]. The differing dynamics of bystander killing between payloads can be used to overcome reduced amounts of target antigen expression to induce tumour response. With increased tumour heterogeneity observed especially in solid tumours, effective cell kill can be induced via bystander mechanisms through potent membrane permeable ADC payloads in low target antigen environments.

The importance of payload selection for stem cell targeting was recently reported in the analysis of two ADCs targeting the Lgr5+ colorectal stem cell and putative cancer stem cell marker [44]. An anti-microtubule targeting anti-LGR5–vc-MMAE ADC was effective in vivo, without affecting the homeostasis of the normal intestinal epithelium in the mouse models explored. In comparison, the DNA damaging anti-LGR5–NMS818 ADC resulted in target antigen-based toxicity that affected the normal intestinal epithelium. Free NMS818 is known to be 1–100 times more potent than free MMAE, increasing the potential for bystander toxicity in Lgr5- non-targeted neighbouring cell populations. This intestinal model exemplifies the important considerations required for the selection of appropriate drug conjugate payloads based on the mechanism of action and the availability of free payload within the tumour type of interest.

## 5. Clinically Approved Antibody–Drug Conjugates

The US Food and Drug Administration (FDA) has approved several ADCs for clinical use in haematological malignancies and some solid tumour types. Many more ADCs are in late stage clinical development and have shown promising initial results (Table 1). The first ADC to gain FDA approval (in 2000) was gemtuzumab ozogamicin (Mylotarg®) for patients over the age of 60 who suffered their first relapse of CD33 positive acute myeloid leukaemia (AML) and were ineligible for chemotherapy [45]. This ADC comprised a humanised IgG4 CD33 antibody coupled with a DNA-binding calicheamicin derivitive. Although this agent sparked renewed clinical interest in the development and design of the ADC as a therapeutic class, this particular agent was voluntarily withdrawn by its manufacture following the post-approval Phase III study, which failed to identify an improved survival within the chemotherapy and gemtuzumab ozogamicin combinational groups when compared to chemotherapy alone with previously untreated AML [46]. Given the heterogeneous nature of AML, it is still believed that a subpopulation of AML patients would benefit from the addition of a gemtuzumab ozogamicin regime. More recent randomised studies have displayed statistically significant overall survival with gemtuzumab ozogamicin with or without chemotherapy [47,48,49,50]. The reason for this discrepancy still remains unclear, but it could in part be due to the lower doses of daunorubicin used in most of these trials or the fractionation of gemtuzumab ozogamicin treatment, which appeared to be better tolerated, allowing for a greater overall dose to be administered. These studies have reignited the clinical debate regarding the clinical use of gemtuzumab ozogamicin [51,52].

In 2011, following an accelerated process, brentuximab vedotin (Adcetris®), received FDA approval for the treatment of patients with Hodgkin lymphoma (HL) after failure of autologous stem cell transplantation (ASCT) or after failure of at least two prior multiagent chemotherapy regimens in patients who are not ASCT candidates. This approval also included treatment for patients with systemic anaplastic large-cell lymphoma (sALCL) after failure of at least one prior multiagent chemotherapy regime [64]. Brentuximab vedotin is comprised of an anti-CD30 chimeric antibody attached to monomethyl auristatin E (MMAE) via a protease-cleavable dipeptide linker [5]. This approval was based on the pivotal Phase II study of 102 patients with an overall objective response rate (ORR) of 75% with complete remission (CR) in 34% of patients. The median duration of response for those patients in CR was 20.5 months; however, showing the veracity of this disease, the median progression-free survival time for all patients on the trial was only 5.6 months [54]. More recently, brentuximab vedotin received additional FDA approval for patents with unfavourable-risk relapsed or primary refractory classic Hodgkin’s lymphoma who have undergone autologous stem-cell transplantation. The AETHERA Phase III trial noted an impressive median progression-free survival (PFS) improvement of 42.9 months in the brentuximab vedotin group compared with 24.1 months in the placebo treated group [65].

The first ADC to receive FDA approval in solid tumours was ado-trastuzumab emtansine (T-DM1, Kadcyla™). T-DM1 was approved for use as a single agent for the treatment of patients with human epidermal growth factor receptor 2 (HER2) positive metastatic breast cancer (MBC) who previously received trastuzumab and a taxane, separately or in combination [66]. T-DM1 is an antibody–drug conjugate comprised of the humanised anti-HER2 antibody trastuzumab (Herceptin®) conjugated to the anti-microtubule cytotoxic, maytnsinoid DM1 via a non-cleavable thioether linker [67]. After the in vitro anti-tumour activity of ado-trastuzumab emtansine in trastuzumab-sensitive and resistant cells lines was demonstrated, as well as efficacy in pre-clinical trastuzumab and lapatinib cross-resistant breast cancer xenograft models [67,68], T-DM1 was well tolerated and showed early signs of efficacy in early phase clinical trials [15,69,70,71]. In the Phase II (TDM4374g) trial, 110 HER2 positive advanced breast cancer patients were compared [72]. These patients had received on average seven therapeutics prior to being selected on the trial, including the HER2 targeted trastuzumab and lapatinib, alongside capecitabine as well as a taxane and an anthracycline [73]. The overall response rate in this patient population was 34.5%, with clinical benefit seen in 48.2% of patients. The key finding from this study was that, even in patient populations that were refractory to trastuzumab and taxanes, a large portion retained HER2 expression and were responsive to a trastuzumab-based ADC. FDA approval for T-DM1 was granted after the successful 991 patient Phase III EMILIA trial in which a progression-free survival overall of 9.6 months with T-DMI versus 6.4 months with lapatinib plus capecitabine was observed [57].

Research into novel ADCs has considerable momentum, with over 100 open clinical trials (clinicaltrials.gov) currently exploring ADCs against novel antigen targets in cancer patients. Although the focus of ADCs has remained in the treatment of haematological malignancies, the success of T-DM1 in solid tumours gives confidence to the clinical development of other novel constructs. A number of ADCs directed against solid tumours are showing promising early results (Table 2).

## 6. Antibody–Drug Conjugate Toxicities

ADCs have been shown to be highly efficacious across a broad spectrum of haematological malignancies and solid tumours in preclinical animal models; however, when evaluated in the clinical setting, their therapeutic windows are hampered by toxicities that are often related to the drug component of the ADC [74]. Dose limiting ocular toxicities have been described for ADCs conjugated with MMAF [75,76]. Similarly, reversible ocular toxicity in the cornea has been reported for DM4-conjugated antibodies [77,78,79]. This appears independent of the target antigen, which does not have significant expression in the eye. The toxicity profile of ADCs conjugated with vcMMAE are similar with adverse events that are the most commonly seen, including acute neutropenia and neuropathy [74]. This has been shown with brentuximab vedotin, with peripheral sensory neuropathy the most common cause of treatment discontinuation [80]. As neurons lack CD30 target antigen expression, it is believed that free MMAE results in an inhibition of axonal transport when the microtubule complex is disrupted. In the pivotal Phase II study, the most serious grades 3 and 4 adverse events were neutropenia (20%), peripheral sensory neuropathy (8%), thrombocytopenia (8%), and anaemia (6%) [54]. Other clinically relevant brentuximab vedotin toxicities relate to infusion-related reactions, diarrhoea, and hyperglycaemia [65].

Much interest awaits the publication of the Phase III inotuzumab ozogamicin INO-VATE trial data. In the Phase II investigation of inotuzumab ozogamicin as a single-agent in patients with indolent non-Hodgkin lymphoma (iNHL) who were refractory to rituximab alone, rituximab in combination with chemotherapy, or anti-CD20 radioimmunotherapy, haematological toxicities were the leading cause of treatment discontinuation. Adverse events of grade 3 or greater were associated were primarily thrombocytopenia (56%) and neutropenia (36%). Non-haematological toxicities were primarily nausea (5%), fatigue (2%), and an elevation of aspartate aminotransferase levels (2%). In this trial, 58% of patients reported an adverse event leading to discontinuation of treatment [81].

For the HER2 targeting T-DM1, thrombocytopenia is the dose-limiting toxicity [73]. In the Phase III clinical trials, thrombocytopenia was observed in 4.7% to 12.9% of patients. The major side effect of concern for the clinical development of the antibody component of T-DM1, trastuzumab, was the initial observation of cardiotoxicity [82]. This is partially explained though due to the high levels of HER2 expression in cardiac tissue. The T-DM1-induced thrombocytopenia appears to be an off-target toxicity. Recent studies have shown that DM1 effects the differentiation and maturation of megakaryocytes from human haematopoietic stem cells. The DM1-meditated cytotoxic effect is less pronounced on mature megakaryocytes [83].

## 7. Recent Antibody–Drug Conjugate Developments

While ADCs have been shown to have therapeutic activity in a range of cancers, there are opportunities to further enhance therapeutic effects with combination therapy, such as combining ADCs with immunotherapy [84]. The ability of a patient’s immune system to co-operate and promote anti-tumour effects has emerged as an important new area of cancer therapy [85]. The induction of tumour cell death following ADC therapy may allow the expression of tumour antigens that, in conjunction with the immune checkpoint blockade, facilitate a host T cell response against the tumour. A recent preclinical study has shown that brentuximab vedotin is a potent inducer of dendritic cell (DC) maturation [86]. This study identified that, in addition to the cytotoxic capabilities of this ADC, brentuximab vedotin can stimulate a host adaptive immune response in both patient and animal models. When combined with PD-1/PD-L1 inhibition, a strong therapeutic synergy was identified.

These adaptive immune studies have been expanded to studies of T-DM1 [87]. It was observed that, in human primary breast tumours, T-DM1 therapy induced an increase in infiltrating lymphocytes (TILs) as well as increased infiltration of effector T cells in a murine breast tumour model. T-DM1, as is the case for brentuximab vedotin, promotes the DC maturation leading to T cell infiltration, while, in combination with anti-PD-1/CTLA-4 therapy, the exhausted T-cell response is reinvigorated. This suggests a strong rationale for the combined use of antibody–drug conjugates with immunotherapy-based approaches.

## 8. Resistance to Antibody–Drug Conjugate Therapy

**Figure 2:**
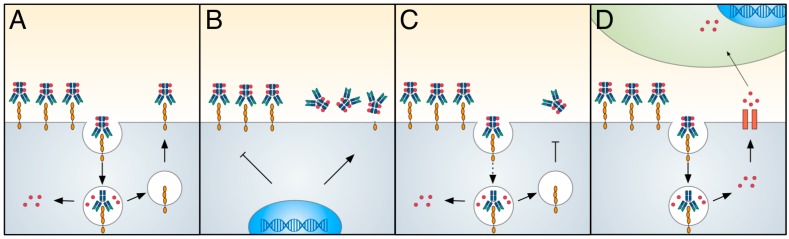
Resistance mechanism for antibody–drug conjugate (ADC) therapies. (**A**) An effective ADC therapy is dependent on high levels of intracellular cytotoxic payload delivery. Multiple mechanisms have been identified which influence the delivery and retention of cytotoxic payloads. (**B**) Reduced antigen on the cell surface can result from reduced target gene expression or presence of increased antigen mutations. (**C**) Reduced cell surface trafficking or recycling will also reduce ADC internalisation. (**D**) ADC payloads are targets for multidrug resistance (MDR) transporter efflux out of the cell, potentially inducing bystander killing effects (payload-dependent).

Under a selective therapeutic pressure, a heterogeneous population of tumour cells will provide the opportunity for the development and expansion of treatment resistance sub-populations. The prevalence of treatment resistance is a challenge for ADC drug development, similar to other cancer therapeutics (Figure 2) [2]. The ability to minimise resistance to ADC therapies is being addressed through multiple approaches, including exploration of mechanisms of resistance in both in vitro and in vivo models, and analysis of tumour samples from clinical trials with large scale sequencing and proteomic approaches.

The mechanisms of resistance to ADCs are complex, and may be influenced by multiple factors. Primary resistance to ADC based therapy may occur. However, even in initially responding patients as treatment continues, as is the case for T-DM1, acquired resistance inevitably develops [57,71]. A critical factor for T-DM1 success is the intracellular concentration of DM1 [88]. Multiple factors influence the amount of internalised cytotoxin, with patients with higher HER2 expression typically display a better clinical response [27]. If the HER2-T-DM1 complex is not internalised, the DM1 intracellular concentration will not reach the cytotoxic concentration required for cell death. The different rates of ADC internalisation have yet to be analysed across tumour types [89]. Once inside the cell, if the lysosomal degradation pathway is impaired or if the endosomal recycling machinery is overactive, then the T-DM1 will avoid degradation or potentially release free DM1 into the extracellular environment, increasing the chances of off-target effects.

Chronic in vivo exposure to ADCs has generated multiple resistance models of interest. Using continuous exposure to trastuzumab-maytansinoid ADCs (TM-ADC), a TM-ADC resistant breast cancer cell lines 361-TM and JIMT1-TM was developed which allowed for proteomic and transcriptional profiling to be performed [90]. This study identified induction of drug transporter ABCC1 and reduction of HER2 antigen expression as key nodes of resistance. The proteomic analysis identified alterations in ubiquitylation, potentially affecting the regulation of vesicle and receptor trafficking. Interestingly, the 361-TM resistant cells had increased sensitivity to the HER2-specific kinase inhibitor neratinib and, quite remarkably, were also sensitive to payload delivery via an alternative cleavable linker. Recently reports have explored the potential of PDX models generated from a metastatic HER2-positive ductal carcinoma with acquired resistance to T-DM1 to shed light into genomic biomarkers for ADC resistance. Exome profiling identified a loss of heterozygosity of the tumour suppressor TP53 coupled with a point mutation, alongside a non-synonymous mutation in HER2 as potential mechanisms of resistance [91].

In genome-wide association studies of breast cancer cell lines following resistance to antimitotic agents such as paclitaxel and monomethyl-auristatin-E (MMAE), a striking breast cancer subtype sensitivity spectrum to these anti-mitotic agents was observed [92]. Basal-like cell lines were significantly more sensitive to both agents than luminal or HER2-amplified cell lines. After RNA interference analysis, overexpression of the ABCC3 drug transporter was critical for the establishment of in vitro resistance. Multidrug resistance (MDR) involves a family of adenosine triphosphate-binding cassette (ABC) energy-dependent transporters who play critical roles in the efflux of cytotoxic compounds from the cell [93,94]. P-glycoprotein (P-gp, otherwise known as ABCB1 or MDR1) can mediate the efflux of several ADC payloads such as calicheamicin, ausristatins (MMAE) and maytansine (DM1) [5,95]. The substrates for P-gp are hydrophobic and thus advances in the design and selection of appropriate linker technologies can prevent active efflux of the ADC payload out of the cell [96]. As such, increases in cellular retention of the maytansinoid DM1 have been observed through the inclusion of a more hydrophilic maleimidyl-based linker compared to non-polar-based linker counterparts [97]. With recent reports identifying Hodgkin’s lymphoma patient samples becoming positive for drug transporters at a time of brentuximab vedotin resistance, further rational ADC linker designs are warranted [98].

## 9. Summary

Antibody–drug conjugates are an exciting treatment strategy for cancer patients. Lessons learned from the development of approved ADCs are informing the selection of novel targets, linkers, payloads, and patient populations most likely to benefit from future ADC-based therapies. With more than 100 clinical trials currently underway, there is great promise for new therapeutic approvals in the future. Importantly, the field must address ways to widen the narrow therapeutic index of ADC-based therapies, which will drive the future development of this class of cancer therapeutics.

## Figures and Tables

**Table 1:** Selected antibody–drug conjugates in clinical development.

Payload	Target Antigen	Antibody–Drug Conjugate	Lead Indication	Phase	Reference
Calicheamicin	CD22	Inotuzumab Ozogamicin	B-cell malignancy	FDA Breakthrough Therapy Designation	[53]
	CD33	Gemtuzumab Ozogamicin (GO)	AML	FDA approved but withdrawn	[46]
DM1	CD22	Brentuximab Vedotin	Hodgkin’s Lymphoma, Systemic ALCL	FDA approved	[54]
	CD56	Lorvotuzumab mertansine	Multiple myeloma	I/II	[55]
	CD138	BT062	Multiple myeloma	I/IIa	[56]
	HER2	Trastuzumab emtansine (T-DM1)	Breast cancer	FDA approved	[57]
	MUC1	SAR-566658	Solid tumours	I/II	[58]
DM4	CD22	Pinatuzumab vedotin + Rituximab	DLBCL, FL	II	[59]
	CD79b	Polatuzumab vedotin + Rituximab	DLBCL, FL	II	[59]
	GPNMB	Glembatumumab vedotin	Melanoma	II	[60]
MMAE	PSMA	PSMA ADC	Prostate cancer	II	[61]
MMAF	EGFR	ABT-414	GBM	IIb/III	NCT02573324 [62]
SN-38	CEACAM	IMMU-130	Colorectal cancer	II	NCT01915472
	Trop2	IMMU-132	Epithelial cancers	I/II	[63]
Liposomal doxorubicin	HER2	MM-302	HER2 positive metastatic breast cancer	II	NCT02213744

**Table 2:** Selected novel antibody–drug conjugates in early development.

Payload	Target Antigen	Antibody–Drug Conjugate	Lead Indication	Phase
Auristatin microtubule inhibitor	PTK7	PF-06647020	Solid tumours	Phase I
	NOTCH-3	PF-06650808	Solid tumours	Preclinical
DM1	CD70	AMG-172	Renal cell carcinoma	Phase I
	CD22	Anti-CD22-MCC-DM1	Non-Hodgkin lymphoma	Preclinical
	Mesothelin	BAY 94-9343	Mesothelioma, pancreatic, ovarian, NSCLC	Phase I
	CD37	IMGN-529	NHL	Phase I
	Folate receptor 1	IMGN853	Ovarian cancer NSCLC	Phase I
	CD56	Lorvotuzumab mertansine	SCLC, Merkel cell, ovarian	Phase I
	CD19	SAR-3419	NHL	Phase I
DM4	Nectin-4	ASG-22ME	Solid tumours	Phase I
	Carbonic anhydrase	BAY 79-4620	Solid tumours	Phase I
MMAE	SLC44A4	ASG-5ME	Pancreatic cancer	Phase I
	SLTRK6	ASG-15ME	Urothelial tumours	Phase I
	CD22	DCDT2980S	Non-Hodgkin lymphoma	Preclinical
	Sodium-dependent phosphate transporter	DNIB0600A	NSCLC, Ovarian cancer	Phase I
	Axl	HuMax-Axl-ADC	Solid, haematological malignancies	Preclinical
	CD19	SGN CD19A	NHL	Phase I
	CD70	SGN-75	RCC	Phase I
MMAF	ENPP3	AGS-16M8F	Renal cell carcinoma	Phase I
	5T4	PF 06263507	Solid tumours	Phase I
PBD	CD19	ADCT-402	NHL	Phase I
	CD70	SGN-CD70A	NHL	Preclinical

## Abbreviations

The following abbreviations are used in this manuscript:

ABC	adenosine triphosphate-binding cassette
ADC	antibody–drug conjugate
ADCC	antibody-dependent cytotoxicity
ALCL	anaplastic large-cell lymphoma
ALL	acute lymphocytic leukaemia
AML	acute myeloid leukaemia
ASCT	autologous stem cell transplantation
CDC	complement-dependent cytotoxicity
CR	complete response
CTLA-4	cytotoxic T lymphocyte-associated protein-4
DAR	drug to antibody ratio
DLBCL	diffuse large B-cell lymphoma
EGFR	epidermal growth factor receptor
EGFRvIII	epidermal growth factor receptor mutant (exon deletion 2–7)
FDA	Food and Drug Administration
FL	follicular lymphoma
GBM	glioblastoma multiforme
GPNMB	glycoprotein-NMB
HER2	epidermal growth factor receptor 2
HL	Hodgkin lymphoma
iNHL	indolent Non-Hodgkin lymphoma
mAb	monoclonal antibody
MBC	metastatic breast cancer
mc	Maleimidocaproyl
mcc	Maleimidomethyl cyclohexane-1-carboxylate, linked to cysteine of mAb
MCC	Maleimidomethyl cyclohexane-1-carboxylate, linked to thiol of cytotoxin
MDR	multidrug resistance
MMAE	monomethylauristatin E
MMAF	monomethylauristatin F
MMTV	mouse mammary tumour virus
NHL	non-Hodgkin lymphoma
NSCLC	non-small cell lung cancer
ORR	overall response rate
P-gp	P-glycoprotein
PABC	Para-aminobenzyloxycarbonyl
PBD	pyrrolobenzodiazepine
PD-1	programmed cell death protein-1
RCC	renal cell cancer
sALCL	systemic anaplastic large-cell lymphoma
SCLC	small cell lung cancer
SPDB	*N*-succinimidyl-4-(2-pyridyldithio) butanoate
T-DM1	Ado-trastuzumab emtansine
TM-ADC	trastuzumab-maytansinoid ADC
TICs	tumour initiating cells
TILs	infiltrating lymphocytes
vc	valine-citrulline
vc-seco-DUBA	valine-citrulline-seco duocarmycin hydroxybenzamide azaindole
